# GALNT2 regulates ANGPTL3 cleavage in cells and in vivo of mice

**DOI:** 10.1038/s41598-020-73388-3

**Published:** 2020-09-30

**Authors:** Xuedan Li, Yiliang Zhang, Minzhu Zhang, Yan Wang

**Affiliations:** Hubei Key Laboratory of Cell Homeostasis, Department of Biochemistry, College of Life Sciences, Wuhan University, Wuhan, 430072 People’s Republic of China

**Keywords:** Cardiovascular diseases, Lipoproteins, Glycobiology

## Abstract

Angiopoietin-like protein 3 (ANGPTL3) is an important inhibitor of lipoprotein lipase and endothelial lipase that plays critical roles in lipoprotein metabolism. It specifically expresses in the liver and undergoes proprotein convertase-mediated cleavage during secretion, which generates an N-terminal coiled-coil domain and C-terminal fibrinogen-like domain that has been considered as the activation step for its function. Previous studies have reported that the polypeptide GalNAc-transferase GALNT2 mediates the *O*-glycosylation of the ANGPTL3 near the cleavage site, which inhibits the proprotein convertase (PC)-mediated cleavage in vitro and in cultured cells. However, loss-of-function mutation for GALNT2 has no effect on ANGPTL3 cleavage in human. Thus whether GALNT2 regulates the cleavage of ANGPTL3 in vivo is unclear. In present study, we systematically characterized the cleavage of Angptl3 in cultured cells and in vivo of mice. We found that endogenous Angptl3 is cleaved in primary hepatocytes and in vivo of mice, and this cleavage can be blocked by Galnt2 overexpression or PC inhibition. Moreover, suppressing *galnt2* expression increases the cleavage of Angptl3 in mice dramatically. Thus, our results support the conclusion that Galnt2 is a key endogenous regulator for Angptl3 cleavage both in vitro and in vivo.

## Introduction

Protein glycosylation is one of the most important posttranslational modification (PTM) that dramatically increases the diversity of protein functions. More than 50% of the proteome are glycosylated in a number of different ways^1^. *N*-linked glycosylation of asparagine (Asn) and *O*-linked glycosylation of serine (Ser) and threonine (Thr) amino acids are the most abundant forms of protein glycosylation^2,3^. *O*-glycosylation may also occur on the tyrosine (Tyr) and hydroxylysine (Hyl) residues^4–6^. In humans, there are several different types of *O*-glycosylation including *O*-GlcNAc, which is mainly found on cytosolic protein^7^, and *O*-GalNAc (mucin-type), *O*-mannose, *O*-xylose, *O*-fucose, *O*-Glucose, *O*-galactose, which are mainly found on proteins passing through the secretory pathway^5,8–12^.

In human, all types of protein glycosylation are initiated by one or two enzymes except the GalNAc type *O*-glycosylation. Human expresses up to 20 isoforms of UDP-GalNAc: polypeptide *N*-acetylgalactosaminyl transferases (GALNTs), which catalyze the initiation step where GalNAc is attached to Ser and Thr residues in proteins. Those GALNTs have different tissue distributions and substrate specificities, with some of them may be partially overlapped^13^. The large amount of GALNTs mediates the site-specific protein GalNAc *O*-glycosylation that can be differentially regulated and play different roles in cellular functions. Among different functions, the site-specific protein GalNAc *O*-glycosylation emerges as an important regulating step for proprotein processing by the proprotein convertase (PC) families^13^.

The mammalian PC is a family of serine proteinases that play central roles in the processing of various protein precursors ranging from hormones and growth factors to bacterial toxins and viral glycoproteins. The PC families includes seven basic amino acid-specific convertases known as PC1/PC3, PC2, furin, PC4, PACE4, PC5/PC6, PC7 and two enzymes processing at nonbasic residues SKI-1/S1P and NARC-1/PCSK9^14–18^. The multi-basic recognition sites for basic amino acid-specific convertases exhibit the general motif (K/R)-(X)_n_-(K/R)↓, where X is any amino acid except Cys and *n* = 0, 2, 4 or 6^19^. The enzyme S1P recognizes the motif (R/K)-X-(L, I, V)-Z↓, where Z is any amino acid except Pro, Cys, Glu and Val. PCSK9 processes its own prosegment at the motif VFAQ↓SIP, with Val being the most critical residue. This prosegment cleavage is critical for PCSK9 secretion and until now no other substrate has been found for PCSK9^19,20^.

*GALNT2* encodes the GalNAc-transferase isoform 2. SNPs in the *GALNT2* locus are strongly associated with triglyceride (TAG) and HDL cholesterol (HDL-C) levels in multi-cohorts of genome wide association studies (GWAS)^21–24^. However, the specific mechanisms of how GLANT2 regulates blood lipid levels are not fully understood. Glycoproteomics studies identified dozes of substrates for GALNT2 and many of them play critical roles in lipoprotein metabolism, such as ANGPTL3, APOC-III, PLTP, APOE and LIPC et al.^25,26^. These proteins either positively or negatively regulate blood lipid levels and the impact of O-glycosylation for their activities remains largely unknown.

Angiopoietin-like protein 3 (ANGPTL3) is one of the best characterized substrates for GALNT2. It specifically expresses in the liver and secrets into circulation that regulate lipid metabolisms mainly through inhibiting lipoprotein lipase (LPL) and endothelial lipase (EL)^27–29^. Loss-of-function mutations in *ANGPTL3* are strongly associated with panhypolipidemia and protect against coronary artery disease in humans^30,31^. ANGPTL3 contains a signal peptide, an extended N-terminal coiled-coil domain (CCD) and a C-terminal fibrinogen-like domain (FLD). During secretion, ANGPTL3 is cleaved at the proprotein convertase processing site (RAPR^224^↓TT) that localizes between the CCD and FLD domains^28,32^. This cleavage is primarily mediated by furin and PACE4^33–35^. The N-terminal domain of ANGPTL3 contains the conserved LPL binding motif and was shown to be more effective in increasing circulating triglyceride (TAG) level when overexpressed in mice^28^. This PC mediated cleavage has been thought as the activation step for ANGPTL3. However, recent study showed that the N-terminal domain or full length ANGPTL3 have similar ability in inhibiting LPL activity with or without its cofactor ANGTPL8 in vitro^36^.

Previous studies showed that ANGPTL3 was glycosylated at Thr^225^ or Thr^226^ by GALNT2, which blocks the PC cleavage of ANGPTL3 in vitro and in cultured cells^26,34^. However, most of these studies were performed in vitro or using reporter system by overexpressing in cultured cells^26,34,37^. In contrast, loss-of-function mutation in *GALNT2* has no impact on ANGPTL3 cleavage in human^38^. Thus whether GALNT2 regulates the cleavage of endogenous ANGPTL3, especially in vivo, remains unclear.

In present study, we systematically characterized the cleavage of Angptl3 and their dependence on GALNT2 and PC both in vitro of cultured cells and in vivo of mice. We first confirmed that the cleavage of Angptl3 is regulated by GALNT2 and PC in cultured cells. Then we checked the cleavage of endogenous Angptl3 in vivo of mice and also in mouse primary hepatocytes. We found that Galnt2 overexpressing or PC inhibition dramatically inhibits Angptl3 cleavage, whereas suppressing *galnt2* expression dramatically promotes its cleavage in vivo. Thus, our results support the conclusion that Galnt2 is a key endogenous regulator of Angptl3 cleavage both in vitro and in vivo. To our knowledge, this is the first report to show that Galnt2 regulates Angptl3 cleavage in vivo.

## Results

### ANGPTL3 undergoes cleavage in vitro and in vivo

**Figure 1:**
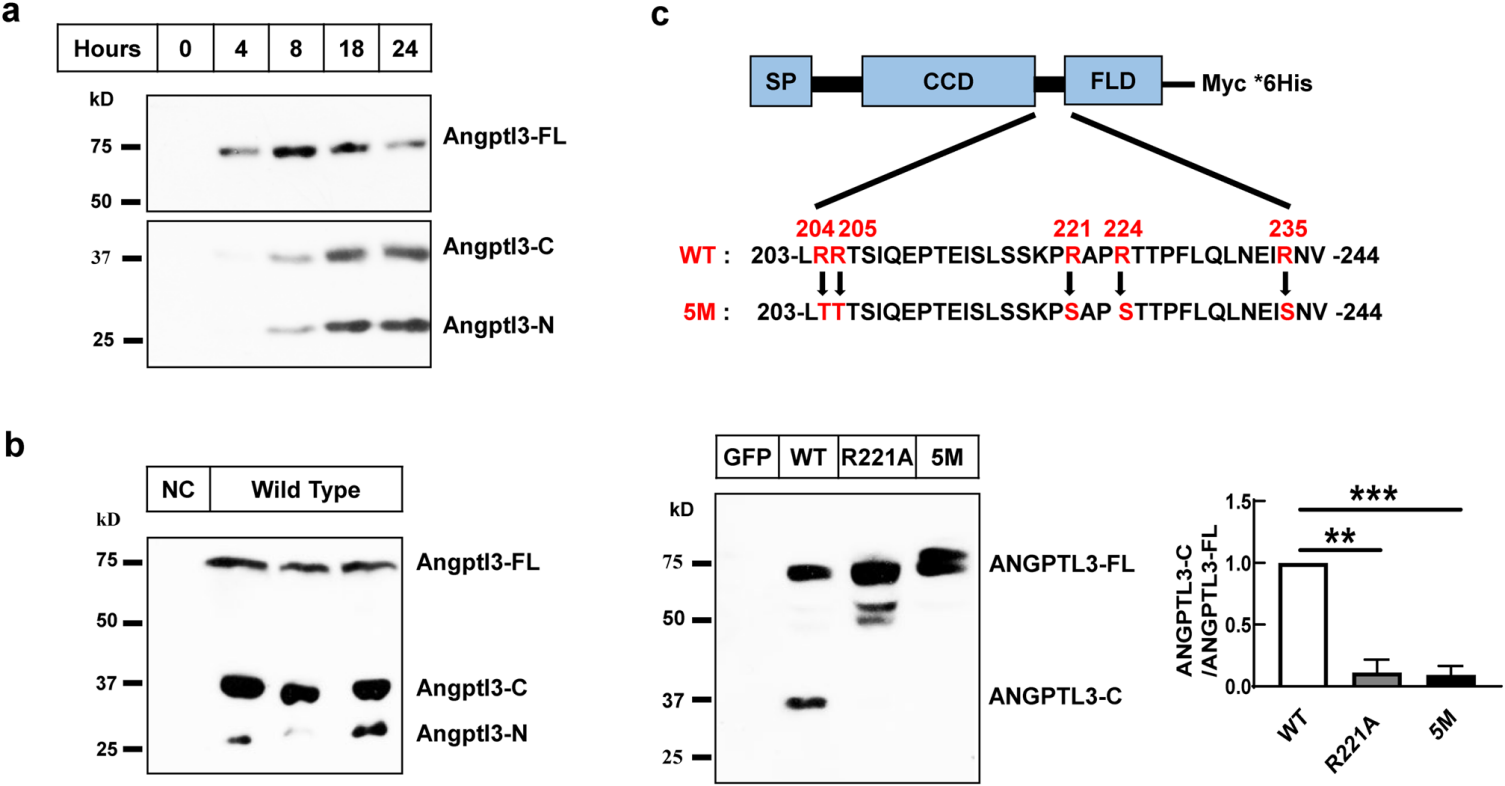
Angptl3 is cleaved in cultured cells and in vivo of mice. (**a**) Angptl3 protein levels in medium of mouse primary hepatocytes. Mouse primary hepatocytes were isolated as indicated in the “Methods”. Mediums were collected at indicated time after plating and were subjected to western blot analysis with a polyclonal antibody that recognize both N terminal and C terminal domains of Angptl3. (**b**) Angptl3 protein levels in serum of mice. Serum was collected at refeeding and subjected to western blot analysis using the same antibody as in A (Male, 8 weeks). NC was serum of *angptl3* knocking down mice and was used as negative control. (**c**) ANGPTL3 protein levels in medium of cultured cells. Hepa1-6 cells were transfected with plasmids expressing the wild-type (WT) or cleavage defective human ANGPTL3 (R221A, or 5M: R204T, R205T, R221S, R224S, R235S) tagged with Myc/His at the C terminal. 48 h after transfection, medium was collected and subjected to western blot analysis with an anti-His antibody. Quantification of ANGPTL3-C versus ANGPTL3-FL immunoblots was shown at bottom right from three independent experiments. SP: signal peptide; CCD: Coiled-coil domain; FLD: Fibrinogen-like domain. ANGPTL3-FL, ANGPTL3-C, ANGPTL3-N stand for full length, C terminal and N terminal domains of ANGPTL3 respectively. Experiments were repeated for at least three times with similar results. **P < 0.01, ***P < 0.001.

ANGPTL3 is specifically expressed in the liver. We first checked the cleavage of Angptl3 in mice and also in primary hepatocytes. We found that the endogenous Angptl3 is efficiently cleaved in both mouse primary hepatocytes and in vivo of mice (Fig. 1a,b). Previous reports have shown that ANGPTL3 is cleaved at the proprotein convertase processing site (RAPR^224^↓TT)^28^. We then tested whether mutation of this site can block the cleavage of ANGPTL3. As shown in Fig. 1c, the wild-type human ANGPTL3 (WT) is efficiently cleaved when overexpressed in Hepa1-6 cells. However, mutant the Arginine at 221 position alone (R221A) or mutant all five Arginine at 204, 205, 221, 224, 235 positions (5 M) completely blocks the cleavage of ANGPTL3 (Fig. 1c), which is consistent with previous reports^28,33^. These results confirm that both endogenous and exogenous ANGPTL3 undergoes cleavage at the proprotein convertase processing site.

### ANGPTL3 cleavage is regulated by Galnt2 and proprotein convertases in cultured hepatocytes

**Figure 2:**
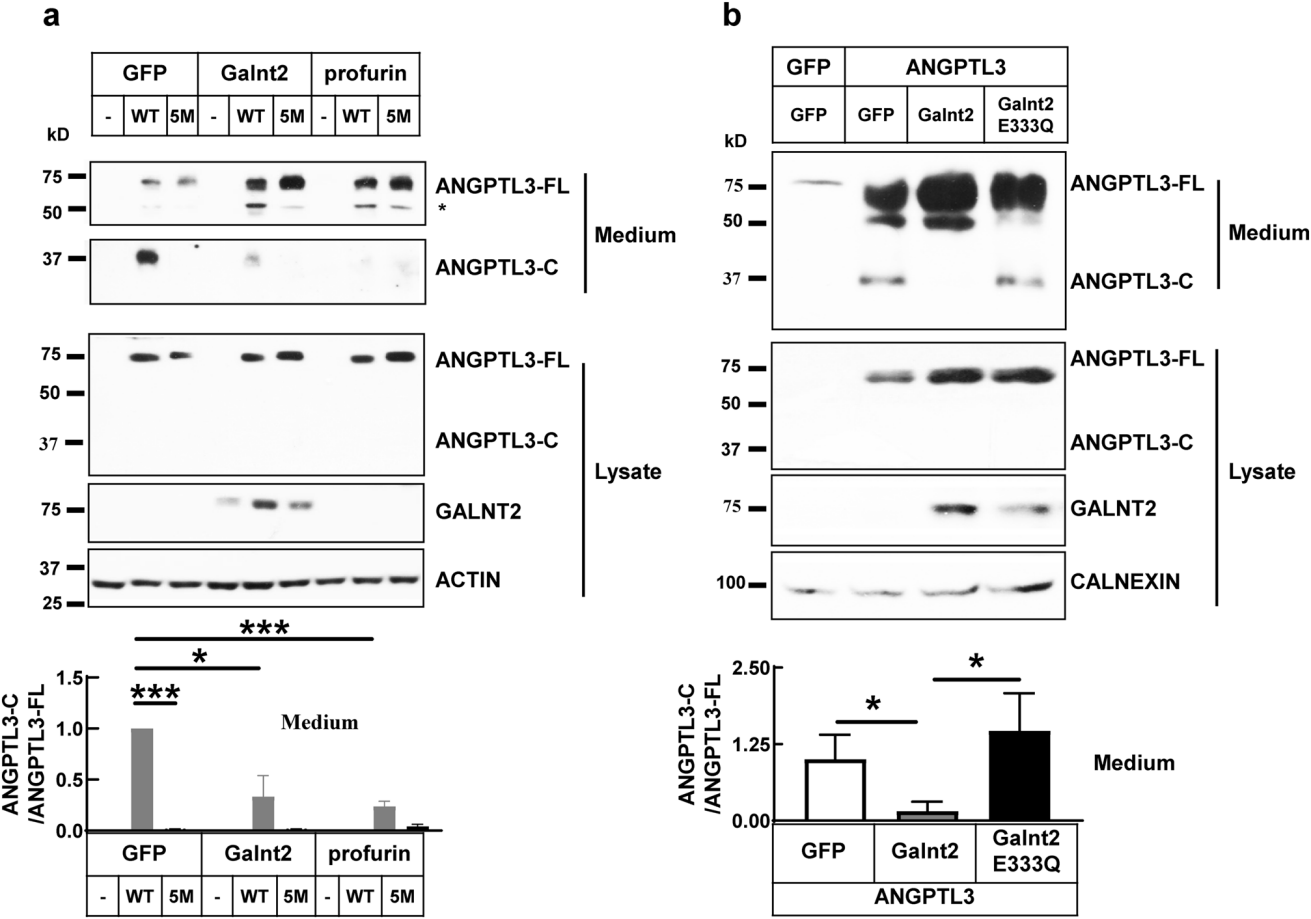
Galnt2 or profurin overexpressing suppresses the cleavage of ANGPTL3 in cultured cells. (**a**) ANGPTL3 protein levels in medium and cell lysate of Hepa1-6 cells that transfected with indicated plasmids. GFP was used as negative control; WT: wild type ANGPTL3; 5M: cleavage defective ANGPTL3 (R204T, R205T, R221S, R224S, R235S). Quantification of medium ANGPTL3-C versus ANGPTL3-FL immunoblots was shown at the bottom from three independent experiments. *Indicates the pre-mature form of ANGPTL3 in cell lysate. (**b**) Enzymatic activity of Galnt2 is required for suppressing ANGPTL3 cleavage. Hepa1-6 cells were transfected with ANGPTL3-Myc/His together with wild-type or enzyme dead mutant Galnt2 (E333Q). Medium was collected and subjected to western blot analysis with anti-His antibody. Quantification of ANGPTL3-C versus ANGPTL3-FL immunoblots was shown at the bottom from three independent experiments. ANGPTL3-FL, ANGPTL3-C and ANGPTL3-N stand for full length, C-terminal and N-terminal domains of ANGPTL3 respectively. Experiments were repeated for at least three times with similar results. *P < 0.05, ***P < 0.001.

ANGPTL3 contains the proprotein convertase consensus site ^221^RAPR^224^ and is cleaved after R^224^ primarily by furin and PACE4. Profurin is the prosegment of human furin and is a strong inhibitor for proprotein convertases. We found that overexpressing profurin strongly decreases the cleaved forms of ANGPTL3 in the medium of mouse hepatocytes Hepla1-6 (Fig. 2a), which is consistent with previous reports^34,35^. Moreover, the 5 M mutation of ANGPTL3, which cannot be cleavage by PC family, is not responsive to profurin overexpression (Fig. 2a, Supplementary Fig. S1). ANGPTL3 is glycosylated at Thr^225^ or Thr^226^ by GALNT2 in vitro and the glycosylation inhibits its cleavage^34^. GALNT2 deficiency increases the cleavage of ANGPTL3 in HepG2 cells^26^. We found that overexpressing wild type Galnt2 significantly decreases the cleavage of ANGPTL3 in the medium (Fig. 2a,b). However, overexpressing the enzymatic dead mutation of Galnt2 (E333Q) has no effect (Fig. 2b), which suggests that Galnt2 suppresses ANGPTL3 cleavage through glycosylation modification^25^. In contrast to the medium, we could not see detectable cleaved forms of ANGPTL3 in cell lysate, which suggests that the decreased cleavage in medium is not due to secretion defect.

### Angptl3 cleavage is regulated by the activity of Galnt2 and PC family in vivo of mice

Humans with homozygous loss-of-function mutations in GALNT2 have decreased glycosylation level at ANGPTL3 (Thr^226^), which suggests that GALNT2 deficiency may lead to increased cleavage of ANGPTL3 in vivo^25^. However, the heterozygous loss-of-function mutation of D314A, which disrupts the enzymatic activity of GALNT2, has no effect on the levels of either full length or N-terminal fragment of ANGPTL3 in human^38^. This leads us to question whether GALNT2 regulates ANGPTL3 cleavage in vivo.

**Figure 3:**
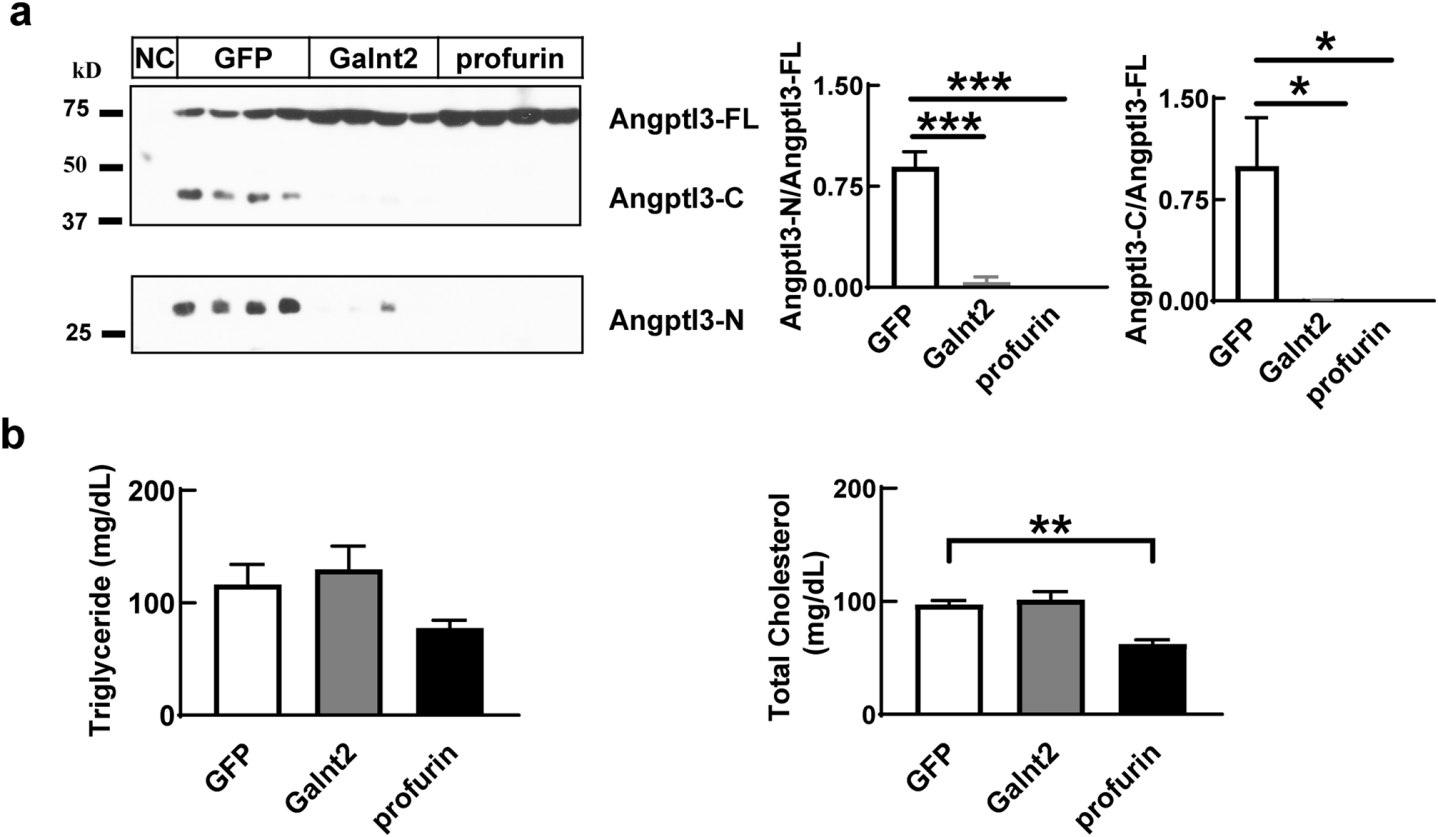
Galnt2 and PC families regulate the cleavage of Angptl3 in vivo. (**a**) Angptl3 protein levels in serum of mice overexpressing Galnt2 or profurin. Adeno-associated virus expressing Galnt2 or profurin were infused into mice through tail vein. 14 days later, serum was collected at refeeding and was subjected to analysis as indicated in “Methods” (N = 5/group, Male, 8 weeks). NC was serum collected from *Angptl3* knocking down mice and was used as negative control. Quantifications of the ANGPTL3-C versus ANGPTL3-FL or ANGPTL3-N versus ANGPTL3-FL immunoblots were shown at the right. (**b**) Serum levels of triglyceride and total cholesterol in mice used in A. Experiments were repeated for at least three times with similar results. *P < 0.05, **P < 0.01, ***P < 0.001.

Consistent with results in cultured cells, overexpressing galnt2 or profurin in mouse liver dramatically decrease the cleavage of Angptl3 in serum (Fig. 3a, Supplementary Fig. S2a), which suggests that both Galnt2 and PC family regulate the cleavage of Angptl3 in vivo. We also checked the Angptl3 protein levels in liver lysate. Consistent with cultured cells, we can only detected full length Angptl3, which suggests that the decreased cleaved forms in the serum are not due to secretion defect (Supplementary Fig. S2b). Mutations of Galnt2 are strongly associated with blood cholesterol levels in human. However, we did not find significant changes for blood TAG level and total cholesterol level in Galnt2 overexpressing mice (Fig. 3b). In contrast, profurin overexpressing significantly decreases the total cholesterol level in mice (Fig. 3b).

**Figure 4:**
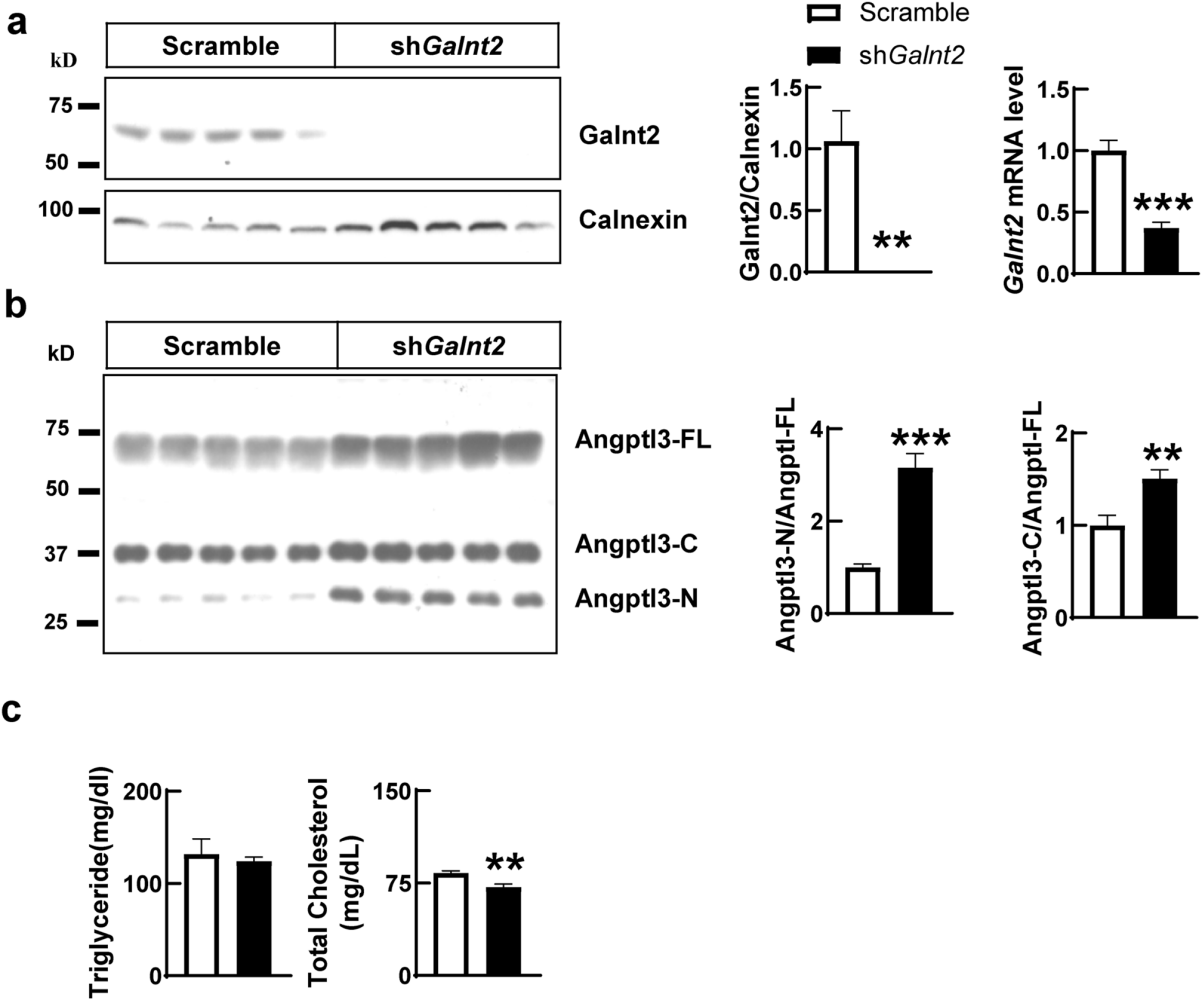
Suppressing *galnt2* expression increases the cleavage of Angptl3 in vivo. (**a**) Hepatic levels of galnt2 protein and mRNA in *galnt2* knocking down or control mice. Wildtype mice were infused with adeno-associated virus that expressing scramble or shRNA against *galnt2*. 14 days later, livers were collected at refeeding and were subjected to analysis as indicated in “Methods” (N = 5, Male, 8 weeks). Quantification of galnt2 immunoblot was shown at the middle. (**b**) Serum levels of Angptl3 in mice used in A. Quantifications of the ANGPTL3-C versus ANGPTL3-FL or ANGPTL3-N versus ANGPTL3-FL immunoblots were shown at the right. (**c**) Serum levels of triglyceride and total cholesterol in mice used in A. Experiments were repeated for at least three times with similar results. **P < 0.01, ***P < 0.001.

Previous report has shown that liver specific *galnt2* knockout mice have normal TAG level but with decreased HDL-C level^25^. To further test whether Galnt2 does regulates blood lipid levels, we knocked down *galnt2* in livers of mice through adeno-associated virus (AAVs) mediated shRNA delivery. As shown in Fig. 4, suppressing *galnt2* expression dramatically increases the cleavage of Angptl3 in serum but not in liver lysate, which suggest that Galnt2 is not only sufficient, but also necessary in regulating Angptl3 cleavage in vivo. Moreover, knocking down *galnt2* in mice does decrease the total cholesterol and HDL-C levels but has no effect on blood TAG level (Fig. 4, Supplementary Fig. S3), which are consistent with previous reports^25^.

## Discussion

Cleavage of ANGPTL3 has long been thought as the activation step for its inhibitory effect on LPL. Previous studies showed that GALNT2 catalyzes the mucin-type (GalNAc-type) *O*-glycosylation on ANGPTL3, which inhibits the PC-mediated cleavage of ANGPTL3 in vitro and in cultured cells^26,34^. However, loss-of-function mutations of *GALNT2* in human have no effect on ANGPTL3 cleavage^38^. In present study, we systematically characterized the functions of Galnt2 and PC family on the cleavage of ANGPTL3. Our results clear demonstrate that both Galnt2 and PC family play critical roles in ANGPTL3 cleavage in both cultured cells and in vivo of mice. The enzymatic activity of Galnt2 is required for suppressing ANGPTL3 cleavage, which suggests that this process requires the glycosylation of ANGPTL3.

Proprotein convertase-mediated cleavage of target proteins ranges from hormones and growth factors to bacterial toxins and viral glycoproteins. Site-specific *O*-glycosylation is emerging as a novel mechanism that regulates the action of PC-mediated processing of proteins^26,34,39^. *GALNT2* has been shown catalyzing the mucin-type *O*-glycosylation on ANGPTL3, APOC-III, PLTP, APOE and LIPC et al.^25,26,34,38^. This mucin-type *O*-glycosylation of ANGPTL3 is on Thr^225^ or Thr^226^, which is close to the PC cleavage site and blocks its cleavage. Our current results, together with previous reports firmly demonstrate that this regulation is sufficient and necessary for ANGPTL3 cleavage both in cultured cell and in vivo of mice.

SNPs in *GALNT2* locus are strongly associated with triglyceride and HDL-C levels in different cohorts of GWAS^21–24^. People with Loss-of-function mutations in *GALNT2* have decreased levels of TAG, HDL-C and to a less extent for LDL-C^25,38^. Genetic disruption of *Galnt2* also leads to decreased level of HDL-C, but with variable changes for TAG, in rodents and nonhuman primates^25^. However, the specific mechanisms of how GLANT2 regulates blood lipid levels are not fully understood. Mechanistic studies suggest that the substrates of GALNT2, including PLTP, APOC-III and ANGPTL3, all contribute to the changes of HDL-C or TAG, but may have species difference^25,38^. Moreover, glycoproteomics studies identified dozens of extra substrates for GALNT2 and many of the target proteins are well known regulators for lipoprotein metabolism, including APOE, APOA1 and LIPC et al.^25,26^. These proteins either positively or negatively regulate blood lipid levels and the functional consequence of O-glycosylation modification on those proteins remains largely unknown. Our current study, together with previous reports, clearly demonstrates that GALNT2 regulates the cleavage of ANGPTL3 in vivo. However, whether this regulation mediates the functions of ANGPTL3, especially on blood lipids regulation, remains to be investigated.

Angptl3 was first discovered as an important player in lipid metabolism through mouse genetics. The mouse strain KK/San has extremely low level of blood TAG and genetic mapping found that the KK/San mice harbor *Angptl3* mutation that produces no detectable protein^27^. Later on, a number of human genetic studies found that mutations in ANGPTL3 are strongly associated with dyslipidemia^22,40–46^. People with loss-of-function mutations in *ANGPTL3* have significantly lower plasma levels of TAG, HDL-C, LDL-C and lower risk for coronary artery disease^31,44,47^. People with complete *ANGPTL3* deficiency have extremely low plasma levels of TAG, LDL-C and HDL-C, without other notable abnormity^30^, which makes it an attractive target for lipid lowering drugs^47,48^.

ANGPTL3 modulates TAG metabolism mainly through inhibiting LPL^42^. LPL is the master intravascular TAG lipase that sitting on the endothelial surface and mediates the clearance of TAG from circulation. ANGPTL3 contains a conserved LPL binding motif at the N terminal and cleavage of ANGPTL3 was long thought as the activation step for LPL inhibition^28^. However, we found that there is no change in blood TAG level in mice, no matter increasing or decreasing the cleavage of Angptl3 by changing the level of Galnt2 or PC activity (Figs. 3, 4). Interestingly, the liver specific *galnt2* knockout mice also have normal TAG level^25^. GALNT2 has dozens of substrates, including ANGPTL3, APOC-III, PLTP, APOE, APOA1 and LIPC^25,26,34,38^. All these proteins play important roles in regulating TAG metabolism. It could be that GALNT2 modifies multiple substrates at the same time and counterbalance each other in regulating blood TAG level. However, recent study also suggests that N-terminal domain of ANGPTL3 and cleavage defective ANGPTL3 have similar ability in LPL inhibition with or without its cofactor ANGTPL8 in vitro^36^.

ANGPTL3 modulates HDL-C and LDL-C metabolism mainly through inhibiting EL^42,49–51^. *Galnt2* knocking down upregulates Angptl3 cleavage dramatically in mice, together with decreased levels of total cholesterol and HDL-C (Fig. 4, Supplementary Fig. S3), which are consistent with previous report^25^. However, whether the decreased cholesterol is caused by increased Angptl3 cleavage remains to be determined. Many of the GALNT2 substrates are well-known regulators of lipoprotein metabolism. New animal models will be needed to dissect the physiological functions for Galnt2 mediated *O*-glycosylation on each substrate. Our finding that Galnt2 and PC do regulate Angptl3 cleavage in vivo is the key step for further mechanistic studies.

## Methods

### Materials

Cell culture medium was obtained from life technologies (Staley Rd Island, USA), and FBS was obtained from Pan seratech (Aidenbach, Bavaria, Germany). EDTA-free protein inhibitor cocktail was purchased from Bimake (Houston, USA), Polyethylenimine (PEI) were obtained from Polysciences (Warrington, PA).

The following primary antibodies were used: Anti-ANGPTL3 antibody (AF136, R&D Systems, Minneapolis, USA), Anti-His antibody (Catalog: M20008F, Abmart, Berkeley Heights, US), Anti-Beta actin antibody (Catalog: 20536-1-AP, Proteintech, Chicago, IL), Anti-Calnexin antibody (Catalog: 10427-2-AP, Proteintech, Chicago, IL). The following secondary antibodies were used: HRP conjugated goat anti-mouse IgG or goat anti-rabbit IgG (Jackson ImmunoResearch), bovine anti-goat IgG (Proteintech, Chicago, IL).

### Mice

All mice are C57BL/6J background. The animal protocols were approved by the Institutional Animal Care and Use Committee of Wuhan University. The authors confirm that all methods were carried out in accordance with relevant guidelines and regulations. Mice were housed in the specific pathogen-free animal facility with controlled environment (12-h light/12-h dark daily cycle, 23 ± 1 °C, 60–70% humidity) at Wuhan University. Mice were fed with standard chow diet unless otherwise indicated. In some experiments, mice were synchronized with 3 days training for food intake by daytime feeding (8:00 a.m. to 8:00 p.m.) and night time fasting (8:00 p.m. to 8:00 a.m.). Mice were sacrificed on day4 at 6 h after refeeding and samples were frozen at − 80 °C or subjected to analysis immediately.

### Cell culture

Hepa1-6 cells were purchased from China Center for Type Culture Collection (CCTCC) and were cultured in high glucose DMEM medium containing 10% FBS and 100 units/ml penicillin G/streptomycin. Cells were infected with lentivirus for transgenes expression. Two days later, cells were changed with serum-free medium. On day 3, medium and cell pellet were collected and subjected to immunoblot or RT-PCR analysis.

Mouse primary hepatocytes were isolated as described previously^52^ and cultured in low-glucose DMEM medium containing 5% FBS and 100 units/ml penicillin G/streptomycin. Three hours after plating, cells were changed with serum-free medium and samples were harvested at indicated time points.

### Plasmids and virus production

Human ANGPTL3 tagged with MYC/His at the C terminal was cloned into pShuttle vectors. The R221A mutant and 5 M mutant (R204T, R205T, R221S, R224S, R235S) were made with QuickChange site-directed mutagenesis according to the manufacturer’s instructions. Mouse galnt2 tagged with Flag at the C terminal was cloned into pShuttle vectors. The enzymatic-dead mutation of galnt2 (E333Q) was made with QuickChange site-directed mutagenesis. Profurin was cloned into pShuttle vectors. The presence of the desired mutation and the integrity of each construct were verified by DNA sequencing. shRNA sequence against galnt2 is: 5′-CGGATGGAGTTGTTGGAATTT-3′. Lenti-virus and adeno-associated virus were produced exactly the same as described before^53^.

### Immunoblotting

Liver or cell pellet were homogenized in lysis buffer (1% v/v TritonX-100, 50 mM Tris-Cl [pH 7.5], 150 mM NaCl, 5 mM EDTA plus protease inhibitor). Protein concentrations in the supernatants were measured using BCA methods. Equal amounts of protein were subjected to SDS-PAGE and immunoblot analysis as previously described^54^. Mouse serum was first diluted for tenfold with sterile saline and then added with sample buffer and heated at 95 °C for 5 min. Equal volumes of samples were subjected to SDS-PAGE analysis.

### Real-time PCR

The real-time PCR were performed as described before^55^. Briefly speaking, the total RNA from mouse liver or cells was isolated by homogenization in Trizol (Sigma, Catlog T9424). cDNA was made from 2 μg of RNA using TaqMan (thermo scientific) with random hexamer primers. Oligonucleotides specific for each transcript were used to amplify by PCR in 2 × SYBR Master Mix (Yeasen, China) according to the manufacturer’s instructions. The mRNAs levels were normalized to the levels of cyclophilin or 36B4. The following primers were used in this study: *Profurin*: forward: 5′-AGGGCCAGAAGGTCTTCACC-3′; reverse: 5′-TTCGTCACTCCTCGATGCCAG-3′; *Galnt2*: forward, 5′-CGCCCTCTGCCTCCCTCTTTC-3′; reverse, 5′-TGATTGCTGCTTGCCCACTTGTTC-3′; *36b4*: forward, 5′-CACTGGTCTAGGACCCGAGAAG-3′; reverse, 5′-GGTGCCTCTGGAGATTTTCG-3′; *cyclophilin*: forward, 5′-TGGAGAGCACCAAGACAGACA-3′; reverse, 5′-TGCCGGAGTCGACAATGAT-3’.

### Blood chemistry

Plasma or serum was collected from the supernatant after centrifugation (4000 rpm X 10 min). In some experiments, plasma lipoproteins were size fractionated by Fast Protein Liquid Chromatography (FPLC) using a Superose 6 column (GE Healthcare). Levels of TAG and cholesterol were measured with commercial kits (Kehua Bio-engineering, Shanghai, China).

### Statistical analysis

Data were analyzed using GraphPad Prism 6 and are presented as the mean ± SEM. Statistical analysis was performed using a 2-tailed Student’s t test. A *P* value less than 0.05 was considered significant.

## Supplementary information

Supplementary Figures.

## Data Availability

The datasets generated during the present study are available from the corresponding authors upon request. Most of the reagents used during these studies are commercially available.
