# Supplementary material for: GALNT2 regulates ANGPTL3 cleavage in cells and in vivo of mice
Source: Sci Rep. 2020 Sep 30;10:16168. doi: 10.1038/s41598-020-73388-3 (PMC7527996; doi:10.1038/s41598-020-73388-3)

GALNT2 regulates ANGPTL3 cleavage in cells and *in vivo* of mice

Xuedan Li, Yiliang Zhang, Minzhu Zhang, Yan Wang*

Hubei Key Laboratory of Cell Homeostasis, Department of Biochemistry, College of Life Sciences, Wuhan University, Wuhan, 430072, People's Republic of China

*To whom correspondence should be addressed: Dr. Yan Wang (Wang.y@whu.edu.cn)

Supplementary Fig. S1

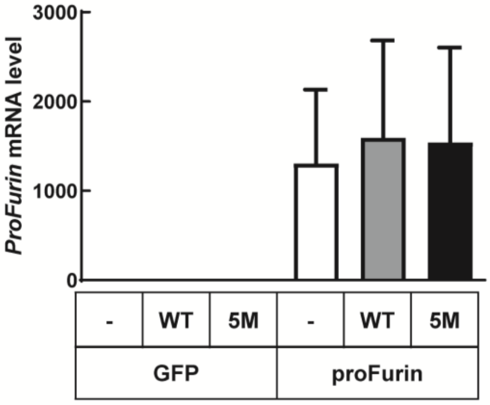

Figure S1: Relative expression levels of exogenous profurin in hepa1-6 cells, corresponding to Figure 2A.

Supplementary Fig. S2

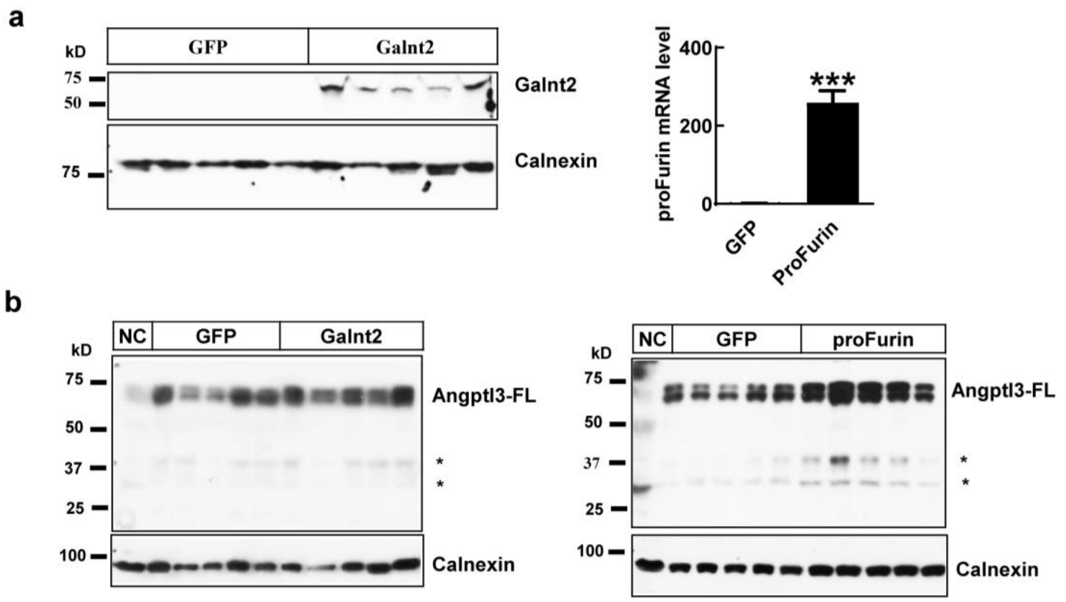

Figure S2: **a**, Levels of galnt2 protein and profurin mRNA in livers of mice used in Figure 3. **b**, Angptl3 protein levels in livers of mice used in Figure 3. *non-specific band. ***<0.001

Supplementary Fig. S3

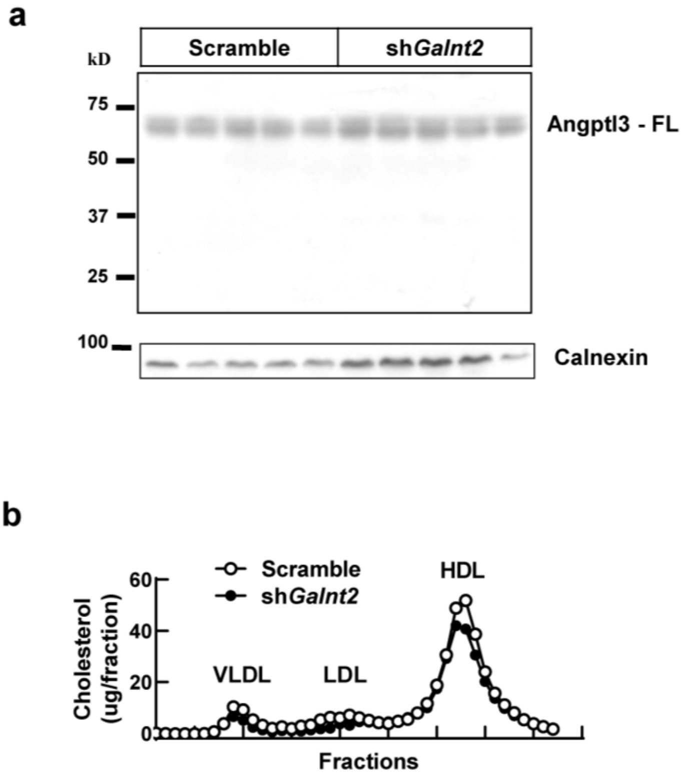

Figure S3: **a**, Angptl3 protein levels in livers of mice used in Figure 4. **b**, FPLC fractionation (Fast Protein Liquid Chromatography) and cholesterol levels in each fractions from plasma of *galnt2* knocking down or control mice used in Figure 4.

Whole gels used in Figure 1

Figure 1a

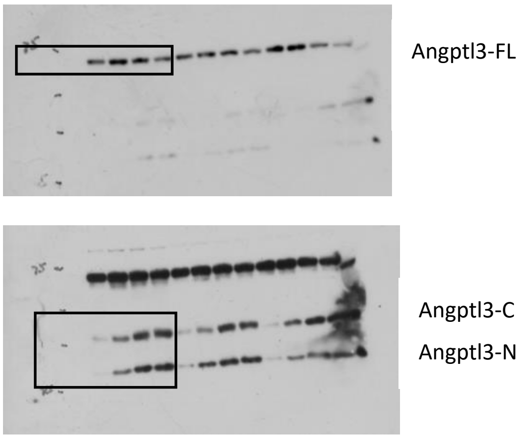

Figure 1b

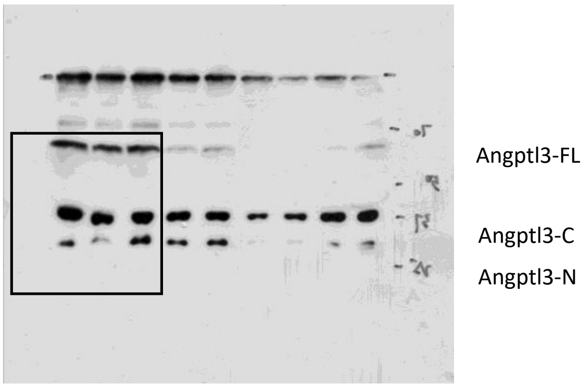

Figure 1c

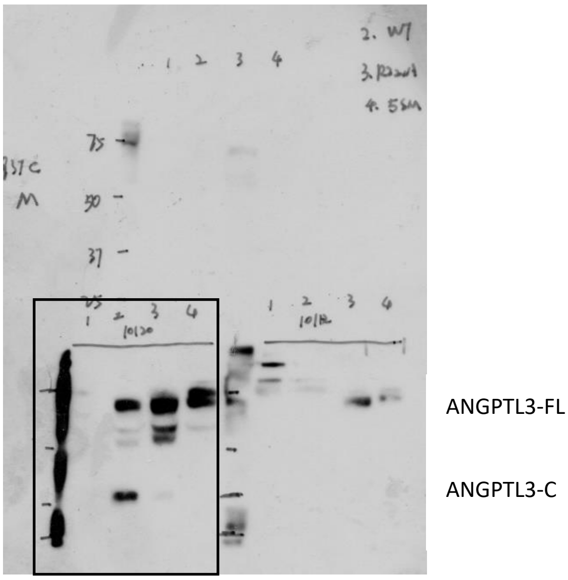

Whole gels used in Figure 2

Figure 2a

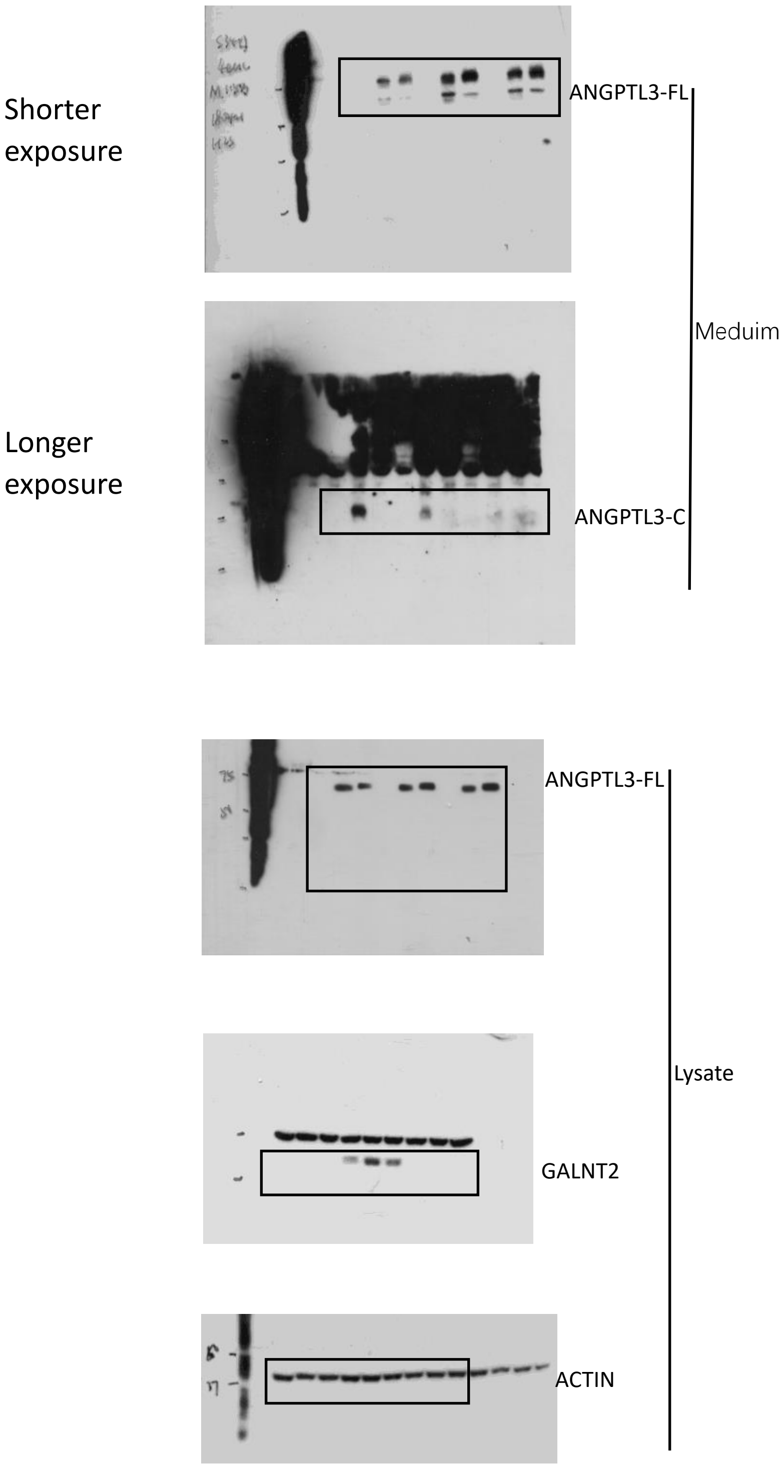

Figure 2b

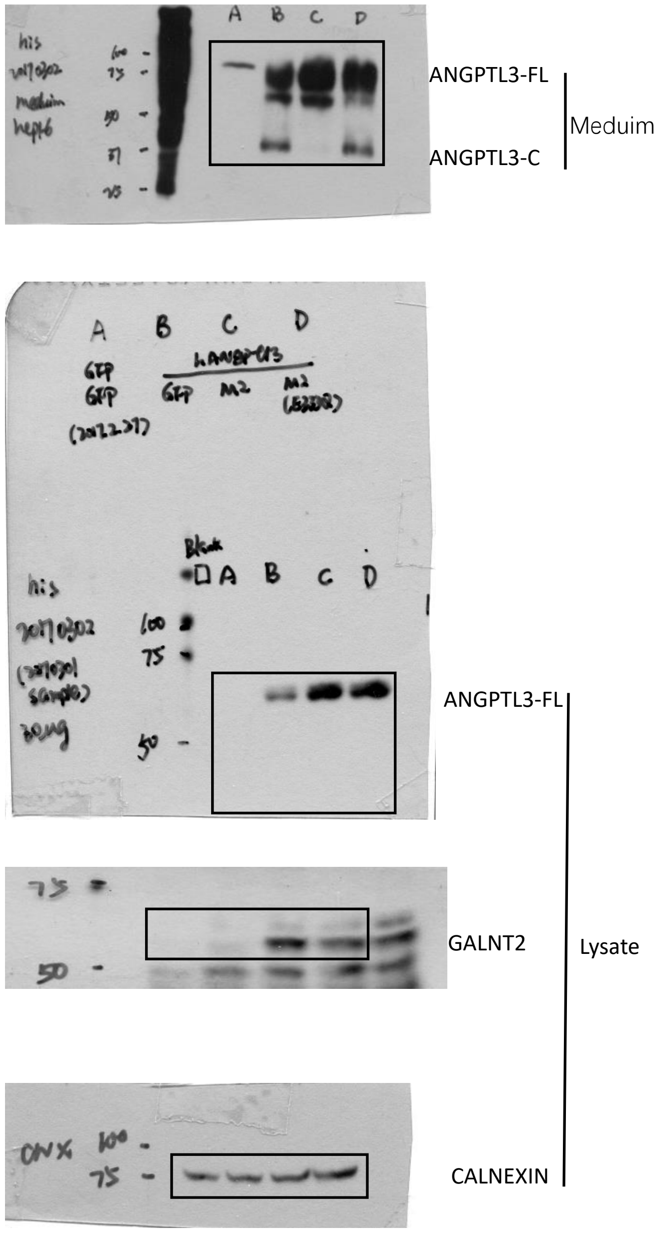

Whole gels used in Figure 3

Figure 3a

Shorter exposure

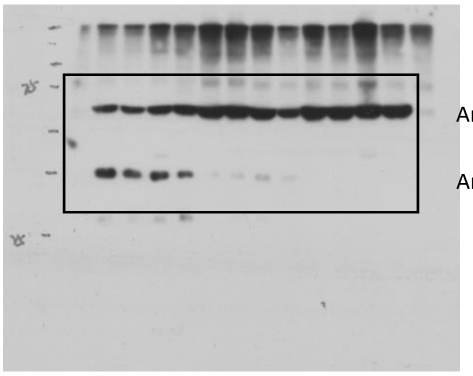

Angptl3-FL

Angptl3-C

Longer exposure

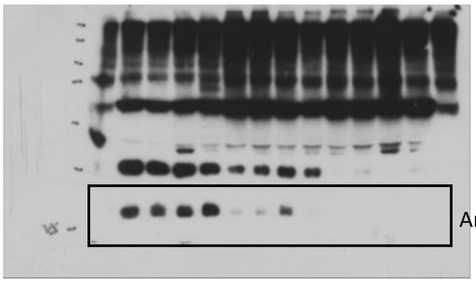

Angptl3-N

Whole gels used in Figure 4

Figure 4a

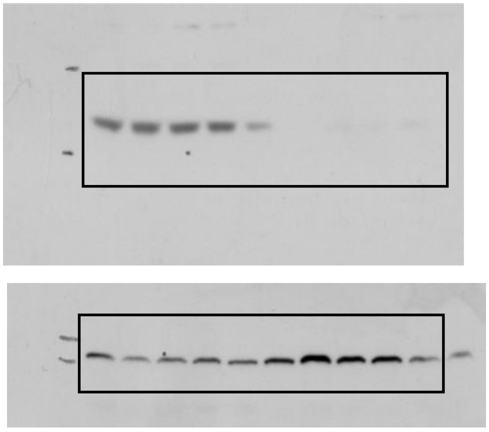

Calnexin

Figure 4b

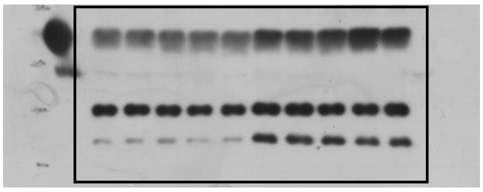

Angptl3-

Angptl3-C

Angptl3-N

Whole gels used in Figure S2

Figure S2a

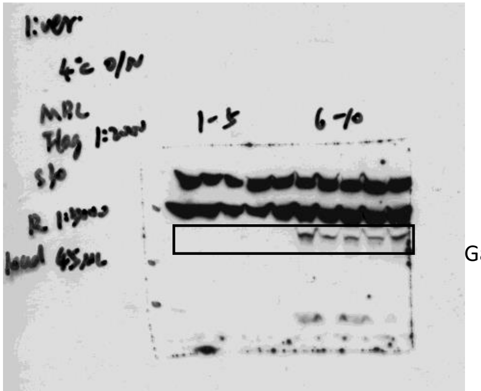

Galnt2

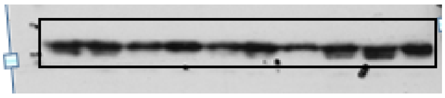

Calnexin

Figure S2b

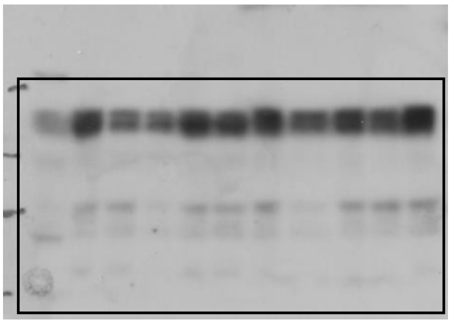

Angptl3 - FL

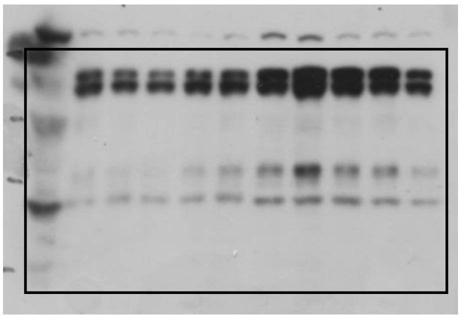

Angptl3 - FL

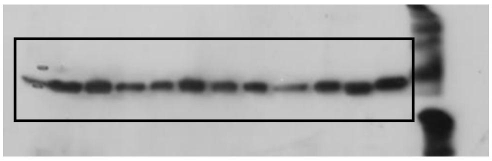

Calnexin

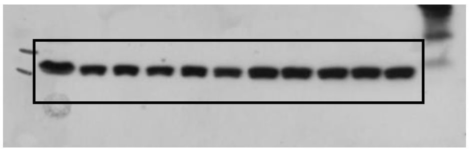

Calnexin

Whole gels used in Figure S3

Figure S3a

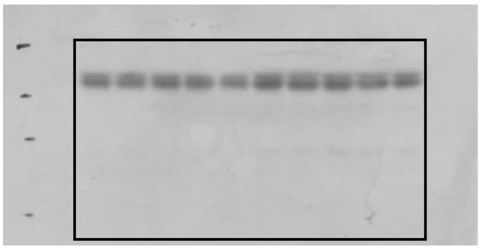

Supplement: Supplementary file 1 — Supplementary Figures. [file 41598_2020_73388_MOESM1_ESM.pdf]
